# Silicon Effects on the Root System of Diverse Crop Species Using Root Phenotyping Technology

**DOI:** 10.3390/plants10050885

**Published:** 2021-04-28

**Authors:** Pooja Tripathi, Sangita Subedi, Abdul Latif Khan, Yong-Suk Chung, Yoonha Kim

**Affiliations:** 1 Department of Applied Biosciences, Kyungpook National University, Daegu 41566, Korea; pooja@knu.ac.kr (P.T.); subedisangu@gmail.com (S.S.); 2 Natural & Medical Sciences Research Center, University of Nizwa, Nizwa 616, Oman; abdullatif@unizwa.edu.om; 3 Faculty of Bioscience and Industry, College of Applied Life Science, SARI, Jeju National University, Jeju 63243, Korea; yschung@jejunu.ac.kr

**Keywords:** image analysis, root morphology, root system architecture, root traits, silicon

## Abstract

Roots play an essential function in the plant life cycle, as they utilize water and essential nutrients to promote growth and plant productivity. In particular, root morphology characteristics (such as length, diameter, hairs, and lateral growth) and the architecture of the root system (spatial configuration in soil, shape, and structure) are the key elements that ensure growth and a fine-tuned response to stressful conditions. Silicon (Si) is a ubiquitous element in soil, and it can affect a wide range of physiological processes occurring in the rhizosphere of various crop species. Studies have shown that Si significantly and positively enhances root morphological traits, including root length in rice, soybean, barley, sorghum, mustard, alfalfa, ginseng, and wheat. The analysis of these morphological traits using conventional methods is particularly challenging. Currently, image analysis methods based on advanced machine learning technologies allowed researchers to screen numerous samples at the same time considering multiple features, and to investigate root functions after the application of Si. These methods include root scanning, endoscopy, two-dimensional, and three-dimensional imaging, which can measure Si uptake, translocation and root morphological traits. Small variations in root morphology and architecture can reveal different positive impacts of Si on the root system of crops, with or without exposure to stressful environmental conditions. This review comprehensively illustrates the influences of Si on root morphology and root architecture in various crop species. Furthermore, it includes recommendations in regard to advanced methods and strategies to be employed to maintain sustainable plant growth rates and crop production in the currently predicted global climate change scenarios.

## 1. Introduction

Silicon (Si) is the most abundant element found in soil after oxygen, and under natural conditions it is generally present as silica (SiO_2_) or as various aluminosilicate forms [1,2]. Depending on the soil type, the Si concentration can range from 25% to 35%, mostly found as quartz, combined with other crystalline silicates (plagioclase, orthoclase, and feldspars)—secondary or clay—and Si-rich minerals (kaolin, vermiculite, and smectite). Most of these Si forms are rarely soluble and difficult to use on a large scale in agriculture [2]. Soluble Si forms in the soil mainly exists as monosilicic (Si[OH_4_]) and orthosilicic (H_4_SiO_4_) acids, which do have a considerable agronomical importance, as plants can readily absorb these two forms. Moreover, the soil chemistry of Si is also determined by soil pH [1,3]: in the pH range of 2–9, Si exists as SiO_2_. However, it can be converted to silicate ions, such as H_3_SiO_4_ and H_2_SiO_4_^2^, when the pH is >9.0 [3]. Even in a soil solution with a pH range of 8–9, Si availability is limited due to the high affinity with soil colloids, especially with iron and aluminum oxides [4]. In addition to soil pH, Si’s bioavailability largely depends on parameters such as soil texture, temperature, organic matter, and accompanying ions [3].

The absorbed Si transports nutrients to the stele and shoot parts, where it accumulates and induces various responses, such as increased plant height, modulation of nutrient uptake, regulation of antioxidant activity and formation of mechanical barriers [4,5,6,7]. In particular, induced mechanical barriers like the cuticle-Si double layer, impede the penetration of pathogens, therefore, preventing disease infections [4]. Another mechanical barrier consisting of Si deposition in the cell wall counteracts invasions by insects and other pests by preventing stylet penetration or tissue chewing [8,9,10]. In the root area, Si promotes root growth and development by favoring the uptake of nutrients and water. Specifically, Si application increases both the fresh and dry weight of roots and their branching angles under heavy metal stress conditions [11]. Furthermore, Si treatments on soybean plants were shown to induce increased root length, diameter, and biomass [12]. In addition to these effects, various changes in root morphology produced by accumulated Si have been reported in many other studies. However, these studies lack consolidative strategies and tools to effectively measure the variations associated with Si application in the root area. Therefore, we investigated the effects of Si on root morphological traits in major crop species and on their root architectures. This review aims also to provide detailed information on the optimal tools and methods to be employed to fully understand the effects of Si applications on phenotypic variation.

## 2. Silicon in Agriculture

Historically, the first important application of Si in agricultural lands was the use of slag (containing a significant calcium silicate component) in paddy cultivation by Japanese farmers in 1920s. With time, it was observed that this substance caused an increase in grain yield and a reduction in the incidence of pathogenic diseases and pest infestations [4]. Such important benefits prompted agricultural scientists to further investigate the role of Si in the cultivation of various crops. The majority of the studies focused on Si occurrence, presence of soluble forms and consequent bioavailability, uptake and transportation, and stress-resistance mechanisms in plants [3,13,14]. Based on the morphophysiological aspects observed in these studies, it was concluded that roots have a major role in Si transportation and absorption [1]. Absorbed Si can move from root to shoot—especially to the leaf area—therefore, its concentration shows a massive variation in the assemblage (0.1–10% range of dry weight). However, Si concentration also depends on the genus and class of plant species [5]; in fact, some plants with high Si contents present unique mechanisms of Si transportation and absorption, regulated by genes, in different parts of the plant [5].

Roots are an essential component of the plant organism, as they provide anchorage for plants growing aboveground, regulate the uptake of water and essential nutrients from the soil, and serve as storage of resources [15]. As previously mentioned, the vast majority of root functions is significantly associated with crop productivity. Therefore, the study of root systems is of considerable interest in agriculture, for determining optimal ways to increase productivity. Roots are investigated from several perspectives: (i) root morphology, (ii) root architecture, (iii) root physiology, (iv) root metabolism, and (v) cellular homeostasis. Of these aspects, root morphology and architecture have been broadly investigated for crop-breeding purposes [1,2].

**Figure 1:**
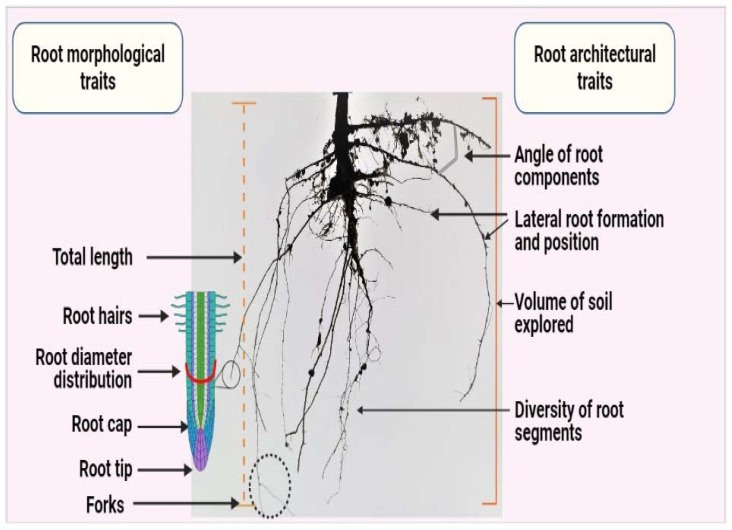
Depiction of morphological and architectural traits of the root system. Currently, various studies focus on the assessment of changes that are due to Si application in the rooting region. Most of these variations are related to either morphological (length, diameter, cap, or hairs) or architectural (root formation and angle of development) traits. Created with BioRender.com.

Root morphology considers a single root axis and surface features, including root diameter, root cap, root hairs, root axis undulation, and the formation patterns of secondary roots (Figure 1) [1]. Root morphological traits are among the most basic traits to be measured. They are important parameters in root research, especially root diameter, specific root length, and root tissue density (Figure 1) [1]. However, these traits represent only a limited number of aspects associated with root function, such as the acquisition of water and nutrients, and they are measured on a two-dimensional (2D) basis [1].

In contrast, root architectural traits—which are analyzed on a three-dimensional (3D) basis—are being prioritized by breeders, as they are involved in the acquisition of multiple mineral nutrients essential for plant growth and productivity in a wide range of environments and different soil conditions. [2]. The target trait considered for root improvement is root plasticity, which regulates the response and adaption to a wide range of environmental stressors [2].

### 2.1. Silicon Transportation and Bioavailability

During the Si uptake processes, the primary and secondary root hairs of a plant absorb Si in the form of silicic acid from the soil and transport it to the cortical cells and then to the xylem [3,4]. When Si reaches the xylem, the silicic acid translocates to shoot parts through the transpiration stream [4]. If silicic acid concentrations exceed 2 mM in the xylem sap, it might be polymerized with other compounds, such as SiO_2_ gel (SiO_2_.nH_2_O), before translocation [13]. The translocated Si is accumulated in different cells [13]. For example, SiO_2_ is deposited in the dumbbell-like vascular bundle cells and in bulliform motor cells in rice leaves, whereas in rice grain, it is primarily deposited in the husk [14].

In relation to the rate of water uptake, three different Si uptake modes have been reported: (i) active uptake, where plants uptake Si faster than they uptake water (rice, wheat, and barley); (ii) passive uptake, where plants uptake Si at the same rate as that of water (oat); and (iii) rejective uptake, where plants try to avoid Si uptake (i.e., exclude Si) [13]. Based on the Si contents present in the shoot (dry weight), plants are arbitrarily classified into three groups: (i) low accumulators (below 0.1% of Si in shoot parts), (ii) intermediate accumulators (around 1% of Si in shoot parts), and (iii) high accumulators (over 5% of Si in shoot parts) [5]. Most of the monocotyledons are high Si accumulators, whereas dicotyledons are primarily intermediate and low accumulators [15]. For example, lowland crops, which are known as monocotyledonous species, show a higher Si accumulation in shoots than dicotyledonous crops—such as cucumber (intermediate accumulator) and tomato (low accumulator)—do [16]. The elevated Si levels in lowland crops are caused by high-density transporters for radial transport and by the transporter for xylem loading [13].

### 2.2. Gene-Regulated Silicon Transportation

Recent studies suggest that Si uptake is mediated by the influx transporter gene, which is responsible for Si absorption from the soil into root cells, and by the efflux transporter gene, which regulates the transport of Si from root cells toward the stele [14]. Si transporters (low Si 1; *Lsi1*) were identified using map-based cloning techniques on mutant rice strains that were defective in Si uptake [3]. This led to further studies of the molecular mechanisms involved in Si uptake in a variety of crops—such as rice, barley, maize, and wheat—to comprehensively understand the dynamics of Si accumulation and transport from soil to stele [5]. Recently, aquaporins found in plant roots were reported to facilitate Si uptake from the soil [5]. The *Lsi1* transporter belongs to the 3-nitro-4-hydroxy-5-iodophenylacetyl (NIP) group (Nodulin-26-like proteins) of aquaporins [6], a class of membrane channel-forming proteins that facilitate passive transportation of water and small uncharged solutes—including silicic acid—from the external solution into the root cells in both dicots and monocots. Their expression patterns and location in cells differ with plant species, but generally they are more expressed in the roots [14,17]. Similarly, the analysis of the transcriptomes of more than 100 plant species, concluded that a plant could directly be classified as “Si-accumulating” or “nonaccumulating”, based on the presence of NIP-III aquaporins [10].

**Figure 2:**
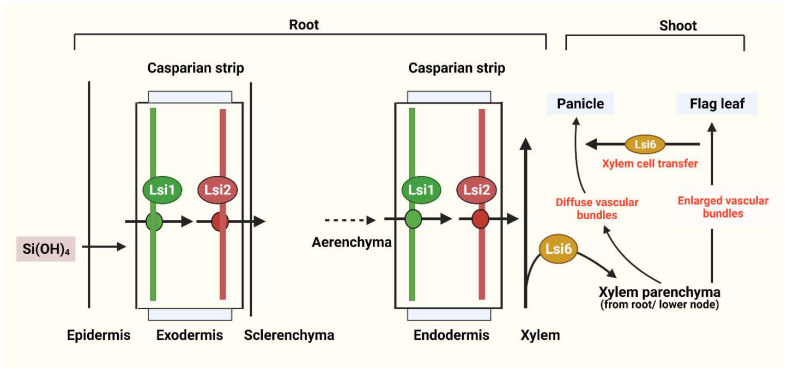
Diagram illustrating Si uptake by roots and its flow through a rice plant (root and shoot), using Si transporters. Lsi1 is localized in the plasma membrane and is responsible for uptake, whereas Lsi2 transports Si into the apoplast across the parenchyma and Lsi6 transports Si from the leaf to the panicles. Modified with reference [18]. Created with BioRender.com.

Subsequently, low Si rice 2 (*OsLsi2*; high affinity Si efflux transporter) and a homologous of *OsLsi1*, named *OsLsi6* (Si influx transporter), were identified [3,18,19,20]. Both transporters (*OsLsi1* and *OsLsi6*), encoded by their respective genes (*OsLsi1* gene and *OsLsi6* gene) for the Si permeable channel, have different roles in the Si uptake process (Figure 2) [21]: the *OsLsi6* gene translocates Si from the xylem to different parts of the plant (Figure 2), whereas the *OsLsi1* gene—known as an influx transporter—was shown to regulate active Si uptake in rice (Figure 2) [22]. Similarly, the role of transporters in other monocot plants, including barley (*HvLsi1* and *HvLsi2*) and maize (*ZmLsi1*, *ZmLsi2*, and *ZmLsi6*), were elucidated. In barley and maize, Si can be absorbed from external solutions through the action of *HvLsi1* and *ZmLsi1* in epidermal, hypodermal, and cortical cells [13]. The absorbed Si is then transported into the endodermis by a symplastic pathway and further into the stele under regulation by the *HvLsi2* and *ZmLsi2* genes [13]. However, in rice, Si is only absorbed at the exodermal cell layer and the *OsLsi2* gene can control its release to the apoplast. The absorbed Si can be transported to the stele from endodermal cells through the expression of *OsLsi1* and *OsLsi2* [13]. The difference in Si uptake and transportation may be due to differences in root structure [14]. For example, in rice, two Casparian stripes are usually present on both the exodermis and endodermis; but in maize and barley, only one Casparian stripe is present on the root endodermis under nonstress conditions [14].

Moreover, due to the presence of the aerenchyma, derived from the lysis of cortex cells between the exodermis and the endodermis in mature rice roots, Si is absorbed into exodermis cells and *OsLsi1* has to be released into the apoplast through the aerenchyma by the *OsLsi2* transporter [14]. *Lsi6* then translocates the absorbed Si from the stele to the shoot through the xylem. Among dicotyledonous plants, as most of them cannot accumulate Si, only a few species are known as Si accumulators [5]. Si transporters were observed in several crops such as pumpkin (*Cucurbita moschata*), cucumber (*Cucumis sativus*, *CsLsi1*, *CsLsi2*), and soybean (*Glycine max*, *GmNIP2-1*, *GmNIP2-2*) [16]. The first Si influx gene among dicot plants was found in the endodermis and root tips of cortical cells in the pumpkin plant (*CmLsi1*), and Si uptake and expression pattern were then observed on two pumpkin cultivars (bloom and bloomless) [23].

Interestingly, Si influx activity was detected in the rootstock of bloom cultivars (*CmLsi1B+*), whereas it was not present in bloomless pumpkin rootstock [23]. Two Si efflux transporters (*CmLsi2-1* and *CmLsi2-2*) were found to be expressed in both the roots and shoots, with no sequence disparity [23]. However, between the two, a major gene responsible for the translocation from the stele to the shoots via the xylem was not identified [15]. Thus, studies that can sufficiently elucidate the mechanisms of Si transport from the root to the shoot in multiple plant species are still not available to date; therefore, this remains an important field of research that requires further investigation.

## 3. The Root System: An Essential Contributor to a Plant’s Life Cycle

The root system architecture (RSA) describes the spatial configuration of roots in the soil by their defining shape, anatomy, and structure [20,24,25]. Through the analysis of the RSA, the soil volume that roots can explore is identified, by determining the length, number, positioning, and angle of each root component [25] and additional aspects are described, such as extending root tips, the formation of lateral roots, and tropism [24,26]. The root system varies between species, within a single species, and even within different root parts in a particular plant, depending on genetic and environmental interactions [24]. Root traits enable the plant to cope with dynamic stress conditions by sensing, adapting, and responding to them [24,26]. For example, root traits associated with maintaining productivity during periods of drought include a small fine-root diameter, deep root growth, and a considerable root length density [27]. Knowledge of the full spatial distribution of roots can help to determine how the soil’s heterogeneously distributed resources are efficiently utilized and, thus, to understand the dynamics of plant productivity [24].

### 3.1. Silicon Effects on Root Morphological Traits

Previous studies have shown that Si application increases leaf width and both shoot and root lengths. In terms of RSA, Si was shown to contribute to an increase in the number of lateral roots in rice (Table 1). Si-treated plants have demonstrated an increase in root traits such as length, diameter, tips, nodulation, and projected area in several crops (Table 1). As the roots uptake Si from the soil and transport it to the shoot area via transpiration stream, Si is deposited in the entire organism [16]. The accumulated Si can stimulate secondary metabolites, such as plant hormones [27,28], which are produced by specific organs and regulate a large number of growth and developmental processes [29]. Auxin, in particular, is a critical regulator of root growth in many crops such as rice, soybean, wheat, and maize [30,31,32,33].

The endogenous concentration of indole-3-acetic acid (IAA) is inversely proportional to the rate of root growth [34]. IAA derepresses gene expression by degrading the gene-repressing proteins via the ubiquitin-mediated proteasome system. Significant production of IAA has not been reported in roots, but the aerial parts supply most of the IAA to the roots. Massive IAA is transported from the shoots to the roots, but the IAA’s influx speed controls its level in the root cells [35]. IAA decelerates root elongation in a wide range of concentrations, whereas it accelerates shoot growth [34]. The effect of auxin in root growth has been documented with supporting evidence of action at genetic and molecular levels [35,36]. IAA-glucosides are a type of IAA conjugates that convert reversibly to free IAA through enzymic hydrolysis. This IAA interconversion to its conjugates is also vital in regulating the IAA level in roots [37,38]. The membrane-located carrier proteins known as auxin resistant 1 (AUX1), multidrug resistant 1 (MDR1), and pin-formed (PIN) are carriers of auxin. AUX1 is an influx protein, the PIN protein is an efflux facilitator, and the MDR1 protein transports IAA and IAA conjugates. Si supplementation promotes an increase in the concentration of both nitric acid and IAA, positively affecting the root’s redox status. In addition, it stimulates the activity of monodehydroascorbate reductase (MDHAR) and dehydroascorbate reductase (DHAR), which are two enzymes of the antioxidant ascorbate–glutathione system, involved in ascorbate recycling [39].

### 3.2. Silicon Mitigates Uptake Or Transport of Heavy Metals in Roots

Industrialization has caused a dramatic increase in the influx of heavy metals, such as cadmium (Cd) and nickel (Ni), into agricultural lands. When farmlands are exposed to these elements, crops absorb them through their roots and translocate them to the shoot parts, especially the grains, resulting in an increased health risk for humans who may consume these contaminated crops. For example, the itai–itai disease was caused by the consumption of rice contaminated with Cd in Japan [49]. Heavy metal toxicity delays plant growth and development by disturbing the cellular functions of proteins, lipids, and elemental components of the thylakoid membranes. Any disturbance caused to the thylakoid membranes and organelles, which are crucial for photosynthetic activities, leads to senescence [50,51]. The effect of Si on heavy metal stress mitigation has been studied in a variety of crops (Table 2). Si application was reported to produce significant biomass and growth improvements in rice plants subjected to heavy metal stress [52]. Nontreated plants suffered severe root damage, whereas Si treatments ameliorated root structure and function. Similar results have been reported in various crops exposed to heavy metal stress (Table 2).

Furthermore, Si treatments were shown to reduce heavy metal uptake, modulating the hormone-signaling pathways involved in host defense and response to stress, such as those of the salicylic, jasmonic, and abscisic acids [52]. The *OsLSi1* and *OsLSi2* genes responsible for Si transport showed significant upregulation of mRNA expression during treatments [52], leading to higher levels of Si accumulation in the roots, which in turn reduced heavy metal absorption. The expression analysis of *OsHMA2* and *OsHMA3* revealed similar results [52].

## 4. Determination of Root Traits

### 4.1. Conventional Methods

Knowledge of plant roots in the past was based on false premises [59,60]. Some of the conventional methods used in plant root studies include the extraction, mapping and in situ imaging methods, and other imaging techniques. In the extraction method, a known volume of soil samples is collected, roots are physically separated from the soil, and rinsed. Then, using stereological or image analysis methods, the root length is measured [61]. The line-intersect method involves manual measurements, where the length is calculated as a product of the number of intersecting roots and a length conversion factor based on the size of the grid used [62,63]. The mapping method is based on recording the occurrence of root contacts on an exposed soil surface. The root contacts detected either on a pit face or core surface are counted with the naked eye or through the inspection of thin polished soil sections under a microscope. These roots are calibrated and measured to convert into length measurements [64].

### 4.2. High Throughput Methods

#### 4.2.1. Root Scanning

**Figure 3:**
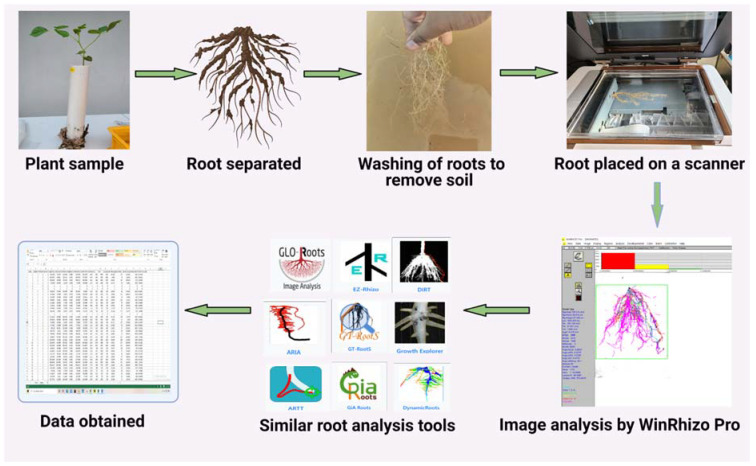
Plant root-analysis methods available for morphological and architectural analysis of roots and applicable to different plants. Methods can range from the use of advanced scanners to image-based root analysis. Created with BioRender.com.

This method requires root samples to be analyzed immediately after harvesting. Samples are firstly washed and then submerged in water within a transparent tray and spread out to maximize separation and minimize root overlapping (Figure 3). Subsequently, the roots are scanned using a general scanner while still being submerged in water in the tray. The scanned root images are analyzed using the WinRHIZO image analysis system (Regent Instruments, Inc., Sainte-Foy, QC, Canada) (Figure 3), which is specifically designed for the measurement of various root forms. It can measure morphology (length, area, and volume), topology, architecture, and color; the software generates graphs illustrating the root diameter distribution and detailing root length, area, and volume of the tips based on root diameter or color function; Furthermore, it displays the analysis of the root image. The root skeleton’s color determines into which diameter class a specific root part is classified. The same color is used to draw the root distribution diagram above the image (Figure 3).

#### 4.2.2. Two-Dimensional Image Method

Several image analysis methods are available, depending on the target trait, such as X-ray imaging [65], magnetic resonance imaging (MRI) [66], 2D imaging [67], and 3D imaging [68]. As 3D imaging techniques are not suitable for application in field studies due to their associated cost, 2D imaging techniques have been increasingly used instead [67]. The acquired root images can be analyzed by a software such as WinRHIZO to examine morphological traits. In the case of leguminous plants, the detection of nodules is complex in field conditions. Therefore, deep learning-based detection and segmentation methods can be employed to accurately determine the size and number of nodules [12].

Adding more validated training data enhances the deep learning-based segmentation network’s performance and can help to process a wide range of data. However, generating annotation images (training data) is inefficient in terms of labor and time because root nodules are tiny and numerous and the amount of training data required for an optimal deep network performance is unknown. Additionally, even with optimally trained networks, segmentation errors can still occur in the analysis of new root images. Therefore, a semiautomatic annotation tool is generally adopted to address these issues. The tool can be customized to generate training data based on other existing tools by setting a simple-to-use and favorable graphical user interface [69]. Furthermore, it is possible to add a function on the existing tool that automatically annotates nodule regions based on our pretrained deep network. This customized tool can also be used for semiautomatic annotation (generating training data) and error correction.

#### 4.2.3. Three-Dimensional Image Method

Comparing the 3D structures of complex root systems allows an improved understanding of their functions [70]. For this purpose, a new system that works with coplanar shadowgrams was introduced: it comprises an object, a fixed camera, and a moving light source [71]. The use of this system reduces the complexity in the calibration step from 6° of freedom (position and orientation of the camera) to 3° (position of the light source), thus, improving the reconstruction results [70].

In this method, a 2D image is obtained for all the images that need to be analyzed. The root systems are grown in containers with gel, which can vary in shape and size based on the type of plant used. These containers are placed on a turntable set, which rotates periodically and allows an image to be captured at each interval. Either the orthographic or perspective projection model can be used for camera calibration. The reconstructions using the regularized visual hull algorithm are compared with those obtained using the conventional visual hull method and expanded versions. To quantify the results, two measures are defined, called the true and false-positive ratios, denoted as tp and fp, respectively, and described as follows:

tp = number of covered silhouette pixels/total number of silhouette pixels

fp = number of covered pixels not in silhouettes/total number of silhouette pixels

The definition of these two parameters highlights the fact that improper 3D reconstruction can cause many false-positive voxels, especially when the shape is thin and delicate. For the visual hull, the fp is always zero. The reconstruction result of the visual hull is expanded uniformly so that the conventional visual hull algorithm can be meaningfully compared. The expansion recovers several missing voxels, but it also increases the fp ratio. When fp = 1, it means that half of the back-projected pixels are incorrect. This confirms that the best results are obtained with the regularized visual hull algorithm, as it increases the tp ratio with only a modest increase in the fp ratio. This is noteworthy, because root structures are thin and delicate; therefore, increasing fp is considerably easier than increasing tp [68].

The main disadvantage of this method is that plant roots have fragile and delicate structures, with very thin branches, which pose a challenge for image-based 3D reconstruction. The inevitable presence of small fractions and possible jittering can cause errors during the image acquisition process. While rotating or moving the container, the gel may cause a slight displacement of roots, resulting in inaccuracy [70]. Furthermore, the software is efficient and works without user intervention, when connected to a 3D reconstruction software [70].

Another imaging technique is magnetic resonance image (MRI), which can generate a structural model of a plant root system using 3D (MRI) data derived from soil-grown plants [72]. The structural model allows the calculation of physiologically relevant parameters; for example, MRI images show the local water content of the investigated sample. The presence of small local amounts of water in roots requires a relatively high resolution, which results in low signal-to-noise ratio images. However, the spatial resolution of MRI images remains coarse, and as fine roots typically have a small diameter, their presence will not be acquired, producing a large number of visible gaps in root system reconstruction. A three-step approach was proposed to reconstruct the root structure: (1) detecting tubular structures, (2) connecting all pixels to the base of the root using Dijkstra’s algorithm, and (3) pruning the tree using two signal-strength-related thresholds [72]. Dijkstra’s algorithm determines each voxel’s shortest path to the root base of the plant, weighing the Euclidean distance by a multiscale “vesselness” measure. As a result, paths running within good root candidates are preferred over paths in bare soil. This method was tested using virtually-generated MRI images of maize and real MRI images of barley roots. In experiments performed on synthetic data, the algorithm presented some limitations in terms of resolution and noise levels [72].

## 5. Conclusions

The effects of Si on root systems has not been extensively studied due to the challenges posed by root phenotyping in field conditions. Although Si plays a major role in improving plant growth, development, and stress aversion in a wide range of climatic conditions, there are still a few examples that explicitly explain the RSA. This is highly desirable since Si is applied to the root zone in most treatments. In this regard, high throughput phenotyping technology, especially image-based phenotyping, has recently been applied to crop research. Studies showed that Si is absorbed by roots as silicic acid and transported to the stele through the transpiration stream in the rhizosphere. It then moves to shoot parts, where it accumulates as SiO_2_ and enhances photosynthetic efficiency by increasing the production of photosynthates, which are essential for plant growth and development. In addition, Si also regulates or stimulates phytohormone synthesis, followed by a series of molecular transcript accumulation in plants. Such biochemical, physiological, and molecular signaling cascades can lead to the activation of processes that ultimately improve root structure and architecture. Generally, root elongation and the enhancement of secondary root traits contribute to plant fitness under stress conditions and can be measured using several methods. However, further research is greatly needed to improve image and data point analysis of the rooting structure. Additionally, modeling and regression methods need to be incorporated to target. Improving machine learning technology can further contribute to an optimal use of these methods for the elucidation of root morphology to achieve goals of sustainability in food production systems.

## Figures and Tables

**Table 1:** Effects of Si application on root morphological traits.

Crops	Concentration of Si Used	Effects on Root Morphological Traits	References
Rice	0.5 mM, 1.0 mM, 2 mM	Si application induced an increase in total root length.	[27]
Soybean	2 mM	Si application during early growth increased root length (tap and lateral roots) and root thickness. Si application increased nodule size and number, increased root projected area, root diameter, and root link diameter.	[12,40]
Barley	0.1 mM, 2 mM	Si application increased root length (ultrastructure modification).	[41]
Sorghum	1.67 mM	Si application induced expansion of the root surface area.	[42]
Chinese liquorice	38.8 mg kg^−1^ soil	Si application significantly increased the root length, root diameter, and lateral root number.	[43]
Alfa-alfa	0.3 g kg^−1^ of soil	Si application increased root volume, number of secondary roots, root biomass.	[44]
Tuberose	200, 400 mg L^−1^ per plot	Si application increased root volume and dry weight.	[45]
Korean ginseng	400 mL Si solution m^2^	Si application increased root production.	[46]
Cowpea	0.96 mM	Si application increased nodulation and nitrogen fixation and led to an increase in the number of bacteroids and symbiosomes per infected nodule.	[47]
Wheat	1.0 mM, 1.5 mM	Si application increased the root length.	[48]

**Table 2:** Effects of Si application on heavy metal translocation in various crops.

Crops		Effects of Si on Heavy Metal Stress Mitigation	References
Mustard	1.5 mM	Si application significantly improved root length and number of lateral roots, under arsenic stress.	[53]
Maize	1.0 mM	Si application induced root growth, under antimony stress Increase in the primary seminal roots, root fresh, and dry weight as well as root branching, under cadmium (Cd) stress.	[11,54]
Rice	1.0 mM	Si significantly improved the growth and biomass of plants and reduced the toxic effects of Cd/copper. Severe damage to root function and structure was avoided.	[52]
Barley	1.0 mM	Si significantly enhanced the activity of antioxidant enzymes in the roots of salt-stressed plants.	[55]
Brazilian ginseng	2.5 mM	Si promoted a significant reduction in deleterious effects produced by Cd in dry weight of roots and shoots.	[56]
Potato	6 L ha^−1^	Si application led to a decrease in the absorption of heavy metals by tubers.	[57]
Soybean	200 mg L^−1^	Si improved physiohormonal attributes and mitigated the adverse effects of salt and drought stress.	[58]

## Data Availability

Not applicable.
